# Within-colony genetic diversity differentially affects foraging, nest maintenance, and aggression in two species of harvester ants

**DOI:** 10.1038/s41598-018-32064-3

**Published:** 2018-09-14

**Authors:** Maya Saar, Pierre-André Eyer, Tal Kilon-Kallner, Abraham Hefetz, Inon Scharf

**Affiliations:** 1 School of Zoology, Faculty of Life Sciences, Tel Aviv University, Tel Aviv, Israel; 2 Department of Entomology, Texas A&M University, College Station, Texas USA

## Abstract

There is accumulating evidence that genetic diversity improves the behavioral performance and consequently the fitness in groups of social animals. We examined the behavioral performance of colonies of two co-occurring, congeneric harvester ant species (*Messor arenarius* and a non-described *Messor* sp.) in fitness-related behaviors, pertaining to foraging performance, nest maintenance, and aggression. We linked these behaviors to the colonial genetic diversity, by genotyping workers, using six and five microsatellite markers for *M*. *arenarius* and *M*. sp., respectively. Correlations of genetic diversity with colony performance and aggression level contrasted between the two species. In *M*. *arenarius*, genetic diversity was correlated with foraging performance and nest maintenance but not with the overall aggression level, while in *M*. sp., genetic diversity was correlated with the overall aggression level, but not with foraging performance or nest maintenance. The two species exhibited similar specific aggression levels, with higher aggression shown towards heterospecifics and lower towards non-nestmate conspecifics and nestmates. However, *M*. sp. workers displayed a tendency to interact for longer with heterospecifics than did *M*. *arenarius*. We speculate that the different foraging strategies, group vs. individual foraging, and possibly also the different mating systems, contribute to the differences found in behavior between the two species.

## Introduction

There has been a growing interest in recent years in the effect of within-population diversity on performance. Elevated population diversity enables specialization and exploitation of a broader range of resources and reduces intraspecific competition^1,2^. Similarly, groups of related individuals often benefit from within-group diversity, because heterogeneous groups possess a larger pool of skills, which better conduce to contending with fluctuating conditions than those of homogenous groups^3^. Such benefits can be reflected in improved survival, foraging, and parental care compared to more homogenous groups^4–6^ (but see^7,8^ for a more complex effect).

In social insects, the effect of within-colony diversity on colony efficiency has been studied mostly in honeybees. Genetic diversity in honeybees contributes to the colony efficiency in performing important tasks, such as nursing and removing carcasses^9,10^. This higher efficiency has led to elevated productivity^11^ and enhanced survival^9,10,12–15^. The mechanism behind this could be that of diversification of the workers’ response thresholds and optimizing task allocation according to colony needs^16–18^. More diverse social insect colonies also forage more efficiently: some ant colonies forage over a longer part of the day^19^, and honey bee colonies exchange more recruitment signals^14,20^ and collect more pollen^21^.

Genetic or phenotypic diversity in ants has been positively correlated with colony growth, success in various tasks, and parasite resistance^22–25^. Colony diversity may also positively affect nest maintenance activities, such as nest reconstruction in ants (as implied in^26,27^) and temperature regulation in bee colonies^28,29^. Similarly, in colonies in which queens mate more than once, some studies have shown a link between patrilines and tendencies to perform specific tasks, plausibly resulting in a more efficient division of labor^26,27,30,31^. Nevertheless, the positive link between within-colony diversity and colony performance is not a general rule and other studies have found no such correlation^32–34^.

The contribution of high genetic diversity to colony performance might be at the cost of increased difficulty in recognizing nestmates. Colonies of social insects must distinguish between nestmates and non-nestmates^35,36^. The failure to do so could lead either to losing nestmates seeking to re-enter the nest or allowing potentially harmful ants to enter the nest (false negative and false positive^37^). Within-colony genetic diversity is expected to decrease aggression towards non-nestmates due to the colony’s low ability to discriminate between nestmates and non-nestmates, because high genetic diversity, existing in many polygynous colonies for instance, makes it harder to form a unique template that does not overlap with those of other colonies^38–41^. However, more recent studies have indicated that the production of a shared communal odor (gestalt odor) in polygynous colonies does not necessarily compromise the colony’s ability to separate between nestmates and non-nestmates, and a unique colony odor is nonetheless produced^42,43^. Plausibly, in such colonies, this could affect aggression levels towards non-nestmates.

In general, attacking heterospecifics is perhaps less common than attacking conspecifics, because the latter are perceived as the greatest competitors. However, in ants, there is evidence that aggression against heterogeneric workers is higher than that against congeneric ones, which is in turn higher than that against nestmates^44^. Workers also increase aggression according to the increasing level of threat expected from each encountered opponent, with the highest aggression directed, for example, towards slave-making ants that seek to steal the colony pupae^45^.

Here, we studied two harvester ant species: *Messor arenarius* and *Messor* sp. Although the latter had previously been considered to be young colonies of a different species, *M*. *ebeninus*, our genetic screening has since revealed it to be a new species, still to be described (the two species are distinguishable; Supplementary Material; Table S1). *Messor*, a granivorous ant genus of medium body size, occurs in the Palearctic region, with the highest number of species in the Mediterranean basin. This genus displays a broad array of foraging strategies, ranging from individual to mass recruitment^46^. The two species studied here are both polymorphic but differ in their average body size, colony size and foraging strategy: while *M*. *arenarius* is mostly an individual forager that sometimes forages in small groups^47–49^, *M*. sp. is a trail-following group forager (Pers. Obs., M.S.). Our first and main goal was to study the correlation between within-colony genetic diversity of the two *Messor* species and foraging performance, nest maintenance, and overall colony aggression. The first two aspects were examined in the field, while the latter was quantified under laboratory conditions, by challenging focal workers with different opponents. Our second goal was to study the specific aggression responses in each species and to determine whether aggression is higher towards heterospecifics than towards conspecifics and nestmates.

For our first goal, we predicted that within-colony genetic diversity would be positively correlated with foraging and nest maintenance performance. Genetically diverse colonies are assumed to produce a more specialized and thus more efficient worker force. In contrast, we predicted that within-colony genetic diversity would be negatively correlated with overall colony aggression level. Regarding our second goal, we predicted that in both species, the specific aggression level would increase with phylogenetic distance and would be the highest against heterospecifics, followed by conspecifics, and the lowest against nestmates.

## Materials and Methods

The experiments were conducted between December 2015 and May 2016 in the Tel Baruch sand dunes (1.5 × 0.5 km area) on the Mediterranean coast in north-west Tel Aviv (32.1283N, 34.7867E; ~20 m above sea level), where *M*. sp. and *M*. *arenarius* are abundant. We first assessed the foraging and nest maintenance performances in the field (9 and 18 colonies of *M*. sp. and *M*. *arenarius*, respectively) over two consecutive days, in order to obtain sufficient data (See Table S2 in the Supplementary material for values obtained during these assessments). We next randomly sampled live workers from both species from the geographically nearest conspecific and heterospecific colonies and transferred them to the laboratory for the aggression assays (237 and 295 workers from 9 and 12 *M*. sp. and *M*. *arenarius* colonies, respectively). Lastly, 103 and 183 randomly sampled workers (from 9 and 15 colonies of *M*. sp. and *M*. *arenarius*, respectively) were stored in absolute ethanol for subsequent genetic analyses in order to determine the genetic colonial diversity and the number of patrilines among the worker force. In all experiments conducted, to ensure the colonies were distinct, they were sampled at 45.6 m ± 36.3; mean ± 1 SD; range [1.6, 128] distance from other conspecific colonies, and were chosen for sampling if they featured only one entrance; we determined there was no second entrance to the nest within a radius of 1.5 meters around the main entrance. For the aggression assays and genetic analysis, workers were collected from the colony entrance. Each sampled colony was then placed in an individually marked and closed plastic container. Air and ground temperatures and relative humidity (hereafter, RH) were measured on each working day (means ± 1 SD: 23.8 °C ± 2.3 °C; 20.8 °C ± 4.3 °C; 31.9% ± 8.3%, respectively). *M*. sp., the currently undescribed new species, was observed in the field for its foraging activity and measured for body characteristics. We report our findings here for the first time.

### Foraging performance and body size

#### Foraging trails, foraging intensity, and colony size

Six colonies per species were observed for their foraging activity in the study area. For the group forager *M*. sp., stable foraging trails were observed and measured using a 50 m measuring tape. Foragers on the trail were counted twice, 10 and 20 min after placing a Petri dish with 2 g of millet seeds 30 cm from the colony entrance. The foraging trail’s fragments of three colonies were photographed as an additional control over possible forager counting error by the observers. For the mostly individual forager, *M*. *arenarius*, foraging trails were not located and foragers were therefore counted twice, in a radius of 1 m around the colony entrance, 25 and 50 min after placing a Petri dish with 2 g of millet seeds 30 cm from the colony entrance. In both species, forager count was performed to estimate foraging intensity and colony size (see Results). Finally, foragers of both species (n = 10) were sampled from the colony entrance in absolute ethanol for morphological identification by a myrmecologist taxonomy expert.

#### Foraging performance test

**Figure 1:**
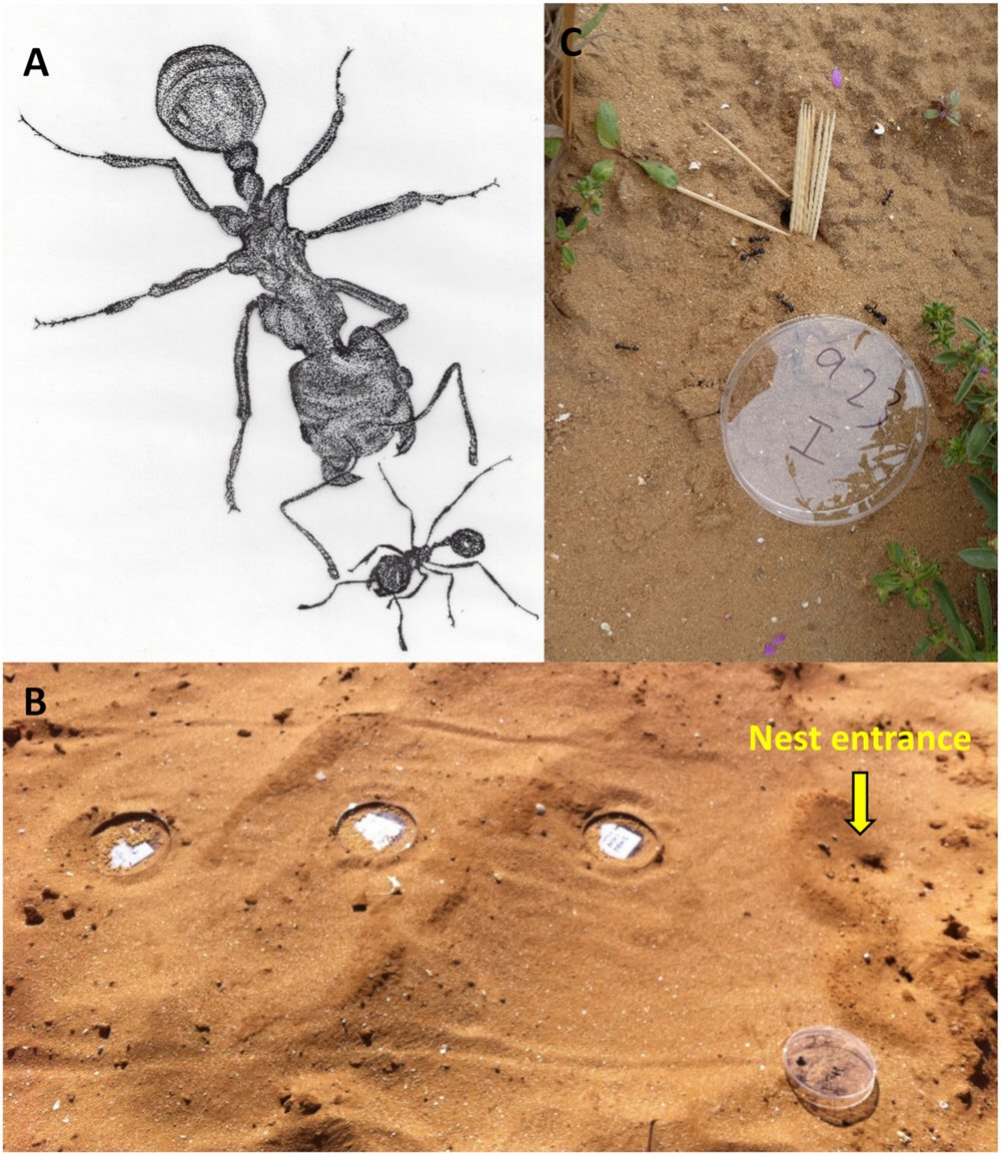
(**A**) An illustration of the aggression assays; *M*. *arenarius* (up) and *M*. sp. (bottom). Workers threaten one another by gaping their mandibles. (**B**) The foraging performance test; plates of millet seeds placed at 30, 60, and 90 cm distance from the colony entrance of *M*. *arenarius*. The marked tracking trail is also visible. (**C**) The nest maintenance test; the colony entrance was blocked with toothpicks, and some were removed by *M*. *arenarius* workers. An additional plate denotes the colony ID. The same procedures were applied to *M*. sp. in both the above (**B**) and (**C**).

Three 9 cm Petri dishes, each with 2 g of millet seeds, were placed concomitantly at increasing distances, 30, 60, and 90 cm from the colony entrance (Fig. 1B). We used three seed plates at different distances concomitantly, because a previous study had indicated that individual and group foragers collect seeds differently. Specifically, the individual forager tends to focus more on the closest patches, whereas the group forager is more flexible regarding the distance of the food patch from the nest^49^. The non-native millet seeds are favored by both studied species (^49^, Pers. Obs., M.S.). *M*. *arenarius* and *M*. sp. were allowed to forage for 50 and 20 min, respectively, before the plates were collected. The test was longer for *M*. *arenarius*, because of their potentially slower depletion of food patches^47,49^. The remaining seeds in all Petri dishes were weighed to the nearest 0.01 mg to estimate foraging performance. Since it was impossible to observe all colonies due to the simultaneous test, tracking trails (a 50 × 100 cm trail that was cleared around each tested colony in the field) were photographed before and after the experiment, to control for tracks of other animals that might have consumed the seeds, as also done in^49^. In only two cases did we spot tracks of birds (probably crows) and we excluded these from the analysis.

#### Body mass and head width

*M*. sp. workers were weighed and their head width measured (n = 60; 10 workers from six colonies). Workers were dried at 60°c for 48 hours and then weighed individually to the nearest 0.01 mg. Heads were photographed with a digital camera (Axiocam ICC5) connected to a stereomicroscope (Stereo Discovery V12, Zeiss, Germany) and measured via ImageJ software^50^. *M*. *arenarius* body mass and head width were already known from a previous study^51^.

### Nest maintenance

The nest entrance of each colony was completely blocked using wooden toothpicks (6.5 cm × 1 mm; Fig. 1C). The number of toothpicks used (between 5 and 33) depended on the size of the nest entrance. Nest entrances were photographed immediately after blocking and 20 min later, and the number of removed toothpicks was counted. In the analysis, we calculated the proportion of toothpicks removed. This method, of measuring nest maintenance by obstructing the nest entrance and allowing the ants to clear it, has been previously applied in other harvester ant studies^52–54^.

### Aggression and interaction assays

Workers of both species were randomly collected from the focal colonies and from the nearest conspecific and heterospecific colonies in the field. Each colony fragment (36 workers sampled) was placed in a round artificial ventilated nest (11 cm in diameter), lined with moist plaster to ensure an optimal level of humidity. Aggression assays were performed under a controlled setting in the laboratory (temperature and RH were 25 °C ± 0.8 °C and 31.3% ± 3.9%; mean ± 1 SD) after six hours of adjustment and up to 24 h post field collection. We placed two workers in a 6 cm Petri dish lined with filter paper to prevent odor transfer between replicates. The ants were first allowed to acclimate, secluded in two glass tubes in the Petri dish for 30 sec. Encounters comprised the focal ant species and one of the three tested opponents (nestmate, non-nestmate conspecific, and heterospecific; Fig. 1A). Each worker was used only once. Heterospecifics were always the other studied species (*M*. sp. when *M*. *arenarius* was the focal species and vice versa). The behavior of the focal ant was then recorded for 3 minutes using the JWatcher software (version 0.9; http://www.jwatcher.ucla.edu), while observing its behavior through a Microsoft LifeCam Cinema HD camera. The focal ant was color-marked with white non-toxic paint, in order to track its behavior. The behavior of the observed focal ant was scored from 0–4 (based on^55^): antennation (0), mandibular threat (1), a threat run (2), short biting with the mandibles (3), abdomen curling and spraying or severely clinging with the mandibles to an organ (4). See Supplementary Material, Figure S1, for pictures of these behaviors. We calculated an aggression index using these scores as follows:

1 $$\frac{\sum _{i=0}^{n}{{A}}_{{i}}\ast {{t}}_{{i}}}{{T}}$$

where *A*_*i*_ and *t*_*i*_ are the aggression score and duration of each interaction, and *T* is the total interaction time (defined as the time in physical contact). We performed 3–12 replicates for each encounter type, the average of which was defined as the aggression index (the value of the equation). This assay enabled us to obtain two measurements: the aggression index and the total interaction time (the denominator of the equation). For *M*. *arenarius*, 12 colonies participated in the encounter types of nestmates, heterospecifics and conspecifics (77, 112, and 106 replicates in total, respectively). For *M*. sp., 9 colonies participated in the encounter types of nestmates and heterospecifics, and 8 colonies participated in the conspecific encounter type (69, 84, and 84 replicates in total, respectively). See Table S3 in the Supplementary material for the aggression assays data, number of workers assigned to each encounter type, the specific aggression indices and the sum of the aggression indices.

### Genetic architecture of colonies

DNA was extracted from ant legs by incubation for 120 min in 100 μl of 5% Chelex at 85 °C^56^ (Bio-Rad, Hercules, CA, USA). Following 3 min centrifugation at 20,000 g, 75 μL of the supernatant were stored at 4 °C. Genotyping was achieved using five and six statistically-independent microsatellite, for *M*. sp. and *M*. *arenarius* respectively, that had been previously developed for other *Messor* species^57,58^. For the current studied species, microsatellite loci were specifically developed under different PCR conditions (see Supplementary Material, Table S4, for details on sets of loci co-amplified and analyzed for each species). For *M*. sp., since it is a new species with an unknown life history, we ascertained that the sampling of nests reflected distinct genetic entities (i.e. colonies). We thus compared the genotypic frequencies of all nests sampled using a log-likelihood (G) based test of differentiation, from GENEPOP ON THE WEB^59^.

Genetic diversity within colonies was estimated as (1) inversely proportional to the genetic correlation between workers, and (2) proportional to the number of matings per queen for each colony^60^. The genetic regression (relatedness, r) between workers of the two species was estimated from 12 worker genotypes (on average) per colony (specifically; mean, range: =11.92, [8, 21]), from 15 and 9 colonies of *M*. *arenarius* and *M*. sp. respectively, using the algorithm of Queller and Goodnight^61^ implemented in the COANCESTRY software v1.0^62^ (http://www.zsl.org/science/software/coancestry). Since the assessed loci showed satisfactory levels of polymorphism, the number of genotyped workers was sufficient to detect differences in relatedness between colonies. For *M*. *arenarius*, the queen and patriline genotypes were inferred from the worker genotypes; each worker was assigned to a given patriline with the maximum-likelihood method implemented in the software COLONY 1.1^63^. Additionally, we calculated the probability of non-detection of an additional male carrying the exact same genotype at all loci studied, using Boomsma and Ratniek’s^64^ equation:

2 $${\boldsymbol{P}}{non} \mbox{-} {detection}={\prod }_{{j}}{\sum }_{{i}}\,{{f}}_{i{,}j}^{2}$$

where *f*_*ij*_ is the frequency of the allele *i* at the locus *j*. For *M*. sp., there were no workers that shared at least one allele at every locus with an inferred queen (see Results) and we were therefore unable to determine the colony patriline number. Workers that could not be unambiguously assigned to the mother queen or to one of the patrilines in each colony (n = 3 from 2 colonies), due to failed PCR amplification or because they shared no allele with the colony queen, were excluded from the analyses. Overall, out of 286 sampled, 284 workers were successfully assigned to their respective colony queen and one of the queen’s respective mates (see Supplementary Material, Table S5, for the genotyping data for both species).

### Statistical analysis

We calculated the following response variables per species and colony: (1) the genetic relatedness, using Queller and Goodnight’s relatedness index; (2) number of patrilines per colony (for *M*. *arenarius* only, see Results); (3) foraging performance: the average of collected seeds (g); (4) nest maintenance: the proportion of obstacles (toothpicks) removed from the colony entrance; (5) colony overall aggression: the sum of the aggression indices for the three encounter types (nestmate, conspecific, and heterospecific); (6) colony aggression index separately towards nestmates, conspecifics, and heterospecifics; and (7) colony total interaction time separately with nestmates, conspecifics, and heterospecifics. The above aggression and interaction parameters (5–7) were obtained using equation number 1.

#### The correlation between relatedness and colony performance and also relatedness and aggression level

To achieve our first goal, we regressed, for each species, the following response variables with within-colony relatedness: (1) foraging performance, (2) colony overall aggression level, (3) colony aggression level towards heterospecifics, (4) colony aggression level towards conspecifics, and (5) colony aggression level towards nestmates. All variables were tested for normal distribution. Variables 3–5 above were square-root transformed (only for *M*. sp.), due to deviations from a normal distribution. In addition, linear regressions were performed using the number of patrilines per colony as the explanatory variable and the above variables 1–5 were the response variables (only for *M*. *arenarius*; see Results). For the nest maintenance proportion variable, we examined whether it correlated with within-colony relatedness (in both species) or patriline number (only in *M*. *arenarius*) using logistic regressions, and we corrected for over dispersion using the quasibinomial option in R (similar to^65,66^).

#### Analysis of specific aggression/interaction responses

To achieve our second goal, we sought to determine whether specific aggression/interaction levels differ between the two species and whether heterospecifics are attacked more frequently than conspecifics or nestmates. We therefore performed two one-way ANOVAs, for each species separately, with encounter type (nestmates, conspecifics, and heterospecifics) as the explanatory variable, and two response variables: (1) the aggression index, and (2) the total interaction time. Both latter variables were averaged per colony. For each species, data were tested for normality and homogeneity of variance. Following, the aggression index variable was square-root transformed due to its deviation from a normal distribution.

## Results

### Foraging performance and body size

#### Foraging trails, foraging intensity, and colony size

For *M*. sp., 1–3 foraging trails per colony were observed, presenting a combined length of 1749.5 ± 547.8 cm; mean ± 1 SD; range [782, 2320], n = 6. All similar reports in the Results section hereafter indicate the mean ± 1 SD. This species initiates foraging on clear vegetation-free trails and seems to follow a pheromone trail; similar to *M*. *ebeninus* (see Supplementary Material, Figure S2A–D). The first count of foragers on the foraging trail was 1218.1 ± 395; range [759, 1707] and the second count was 1328.5 ± 397.6; range [857, 1806]. Controlling for possible counting error of the foragers via photos of three colonies’ foraging trails revealed an average of 86.5% accuracy in field counting, indicating that counting in the field was relatively reliable. For *M*. *arenarius*, the first count was of 25.8 ± 8.9 foragers; range [15, 36] and the second count was 34 ± 11.9 foragers; range [17, 47]. In mostly laboratory studies, the percentage of foragers out of the total colony is between 10–14%^67–70^.We therefore estimated colony size based on its forager number. Thus, a colony size of *M*. sp. could be ~12,700 workers, while in *M*. *arenarius* it could be ~300 workers. Our method seemingly works better for the group forager *M*. sp. than for the individual forager, because the former’s foraging trails are detectable. Colony size for *M*. *arenarius* usually ranges between 800-1,500 individuals (references within^71^), although another study suggests that *M*. *arenarius* colonies consist in up to 5,000 workers, but no measurement methods are provided^72^.

#### Foraging performance test

Foraging performance, expressed as the average of seeds collected from the three plates, within the given time period, was 0.608 ± 0.671 g; range [0.02, 2.00] for *M*. *arenarius*, and 0.289 ± 0.255 g; range [0.01, 0.61] for *M*. sp. The mean ± 1 SD mass of one millet seed is 5.6 ± 0.8 mg (n = 100) and, accordingly, the total number of seeds taken by the ants was estimated at 120 ± 127.3 g; range [4, 355] for *M*. *arenarius* and 51.3 ± 45.2 g; range [1, 107] for *M*. sp. Moreover, since each ant can carry only one seed at a time, the number of seeds taken is equivalent to the number of trips made.

#### Body mass and head width

The dry body mass of *M*. sp. workers was 1.072 ± 0.466 mg; range [0.43, 0.251] and head width was 1.286 ± 0.199 mm; range [0.909, 1.879]. *M*. *arenarius* dry body mass was 42 ± 15.45 mg and head width 3.13 ± 0.58 mm (an average from two localities in Israel^51^).

#### The correlation between relatedness and foraging performance

**Figure 2:**
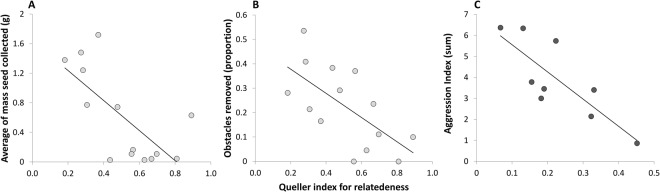
The negative correlation between *M*. *arenarius* colony genetic relatedness and (**A**) foraging performance (average of seed collection), and (**B**) nest maintenance (proportion of obstacle removal), in bright gray dots. (**C**) The negative correlation between *M*. sp. colony genetic relatedness and colony overall aggression (the sum of the aggression indices for the three encounter types), in dark gray dots. Note that the Y axis scale differs from the scale in Fig. 3A, because here we present the sum of aggression levels during the different encounter types.

Foraging performance in *M*. *arenarius* increased with decreasing relatedness, i.e., with increasing genetic heterogeneity (F_1,12_ = 11.402, P = 0.005, R^2^ = 0.487; Fig. 2A). However, the number of patrilines in *M*. *arenarius* was not correlated with foraging performance (F_1,12_ = 2.59, P = 0.13, R^2^ = 0.18). In *M*. sp., relatedness was not correlated with foraging performance (F_1,7_ = 1.37, P = 0.27, R^2^ = 0.16).

### Nest maintenance

#### General values

Nest maintenance, measured as the proportion of obstacles removed from the nest entrance, was 29% [0, 83%] and 12% [0, 45%] (mean and range) for *M*. *arenarius* and *M*. sp., respectively.

#### The correlation between relatedness and nest maintenance

In *M*. *arenarius*, nest maintenance increased with decreasing relatedness, similar to foraging performance (N = 14, t = −2.737, P = 0.018; Fig. 2B). Moreover, the correlation between the number of patrilines and nest maintenance showed a positive, marginally non-significant trend (N = 14, t = 2.142, P = 0.053). In contrast, in *M*. sp. there was no correlation between relatedness and nest maintenance (N = 9, t = −0.63, P = 0.54).

### Aggression and interaction assays

#### The correlation between relatedness and aggression

In *M*. *arenarius* overall aggression level was not correlated with within-colony genetic relatedness or patriline number (F_1,10_ = 0.43, P = 0.52, R^2^ = 0.04; F_1,10_ = 0.74, P = 0.41, R^2^ = 0.06, respectively). Specific aggression levels were also not correlated with relatedness in *M*. *arenarius* (aggression towards conspecifics: F_1,10_ = 0.21, P = 0.65, R^2^ = 0.02; heterospecifics: F_1,10_ = 0.74, P = 0.41, R^2^ = 0.07; and nestmates: F_1,10_ = 0.09, P = 0.76, R^2^ = 0.01). The same pattern held true for the patriline number (aggression towards conspecifics: F_1,10_ = 0.004, P = 0.94, R^2^ = 0.0004; heterospecifics: F_1,10_ = 1.04, P = 0.33, R^2^ = 0.09; and nestmates; F_1,10_ = 0.41, P = 0.53, R^2^ = 0.03). In contrast, in *M*. sp., overall aggression decreased with increasing within-colony genetic relatedness (F_1,7_ = 13.365, P = 0.008, R^2^ = 0.656; Fig. 2C). Specific aggression levels towards heterospecifics also decreased with increasing relatedness (F_1,7_ = 6.748, P = 0.035, R^2^ = 0.491), but correlations with the other specific aggression levels were not significant (aggression towards conspecifics: F_1,7_ = 3.46, P = 0.11, R^2^ = 0.33; and nestmates: F_1,7_ = 0.11, P = 0.76, R^2^ = 0.01). All correlations of specific aggression levels with relatedness for both species are presented in the Supplementary Material (Figure S3A–F).

#### Analysis of specific aggression/interaction responses

**Figure 3:**
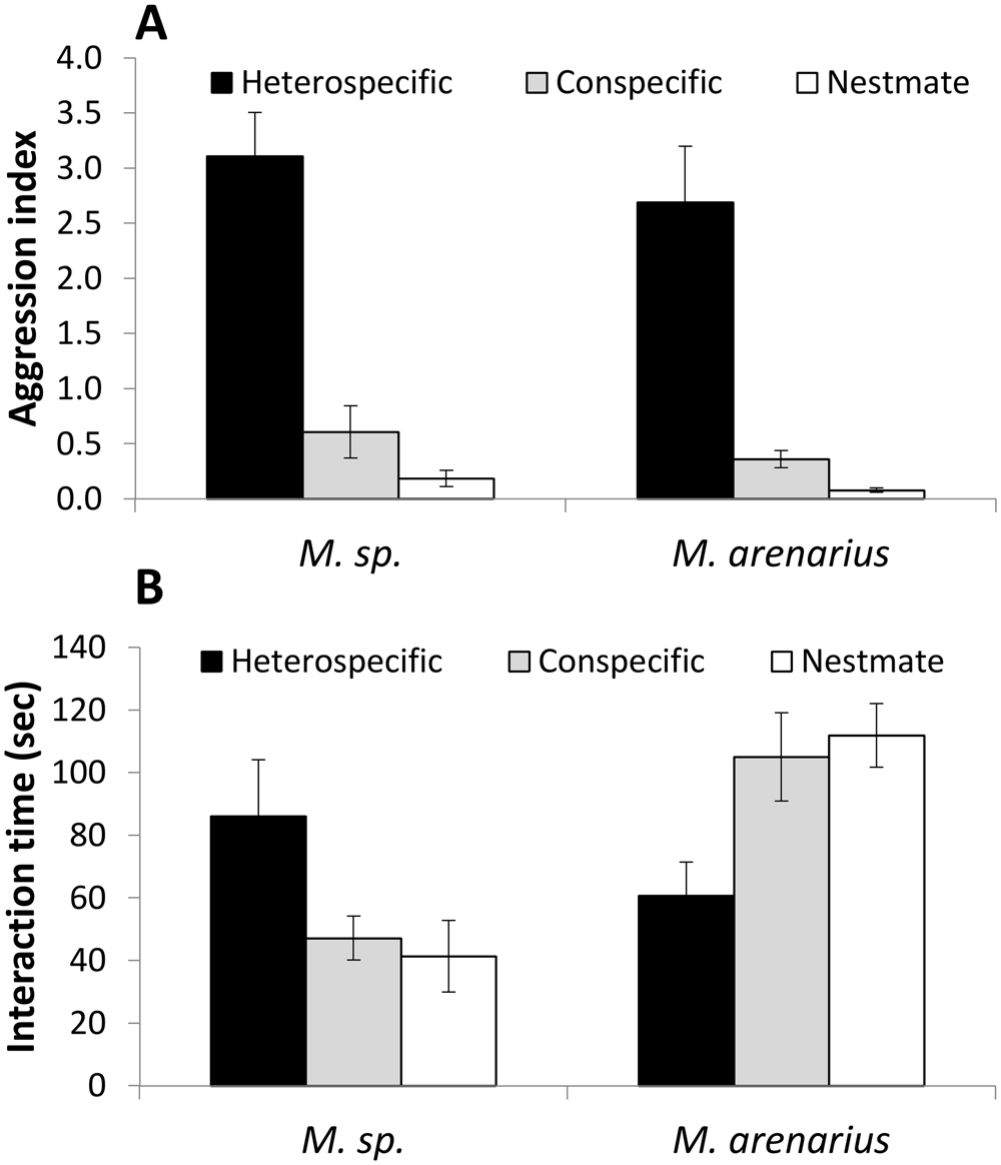
(**A**) The aggression index of *M*. sp. and *M*. *arenarius* (mean ± 1 SE) in the three encounter types; heterospecific (black), conspecific (gray), and nestmates (white). (**B**) The interaction time of *M*. sp. and *M*. *arenarius* (mean ± 1 SE) with; heterospecifics (black), conspecifics (gray), and nestmates (white).

Workers of both species were more aggressive towards heterospecifics than towards conspecifics and nestmates (*M*. *arenarius*: F_2,33_ = 65.453, P < 0.001; *M*. sp.: F_2,24_ = 27.199, P < 0.001; Fig. 3A). Regarding total interaction time, *M*. *arenarius* spent less time interacting with heterospecifics than with conspecifics and nestmates, whereas *M*. sp. tended to spend more time interacting with heterospecifics than with conspecifics and nestmates (F_2,33_ = 5.073, P = 0.011 and F_2,24_ = 3.119, P = 0.062 respectively; Fig. 3B). All Tukey post-hoc comparisons: P < 0.05.

### Genetic architecture of colonies

The genetic analysis for *M*. sp. colonies revealed: (1) All colonies sampled were distinct genetic entities, i.e., distinct colonies (*G-test* for genotypic differentiation; P < 0.001). (2) At least one maternal allele at every locus was not shared by the workers, suggesting the occurrence of more than one reproductive queen. Consequently, we could not unequivocally infer patrilines. The apparent occurrence of multiple reproductives in this species resulted in relatively low relatedness indices among workers (i.e., high genetic diversity), ranging from 0.067 to 0.451 (mean ± 1 SD; 0.228 ± 0.119). In contrast, in all of the *M*. *arenarius* colonies, a single queen was inferred. Moreover, the queens were facultatively polyandrous and had mated with 1 to 3 males (mean ± 1 SD; 1.93 ± 0.79; 5 queens out of 15 were singly mated). This result was robust and unlikely to have been biased to the possibility of non-detection of an additional male carrying the same alleles at all loci, as this probability is low for all males inferred (equation number 2; *P* non-detection < 0.0037). This mating system resulted in higher relatedness indices in *M*. *arenarius*, varying from 0.182 to 0.891 (mean ± 1 SD; 0.521 ± 0.211), compared to *M*. sp. (see Supplementary Material, Table S2, for the number of workers genotyped, Queller relatedness indices in both species and patriline number in *M*. *arenarius*).

## Discussion

Within-colony genetic diversity often contributes to the colony overall performance. Our results, however, are more complex, and differ between the two studied ant species. First, we predicted that within-colony genetic diversity would be positively correlated with foraging and nest maintenance performance. While the more genetically diverse colonies of *M*. *arenarius* maintained their nests better and foraged more extensively, there was no such link in *M*. sp. Second, the overall colony aggression of *M*. sp. was positively correlated with its genetic diversity – diverse colonies were more aggressive – but there was no such link in *M*. *arenarius*. However, we predicted that overall colony aggression level would be negatively correlated with within-colony genetic diversity, in contrary to our findings, and we later offer several suggestions to explain this result. We also raise several possible explanations for the differences detected between the two species, while acknowledging that the comparative method may be a complementary tool when tackling this question. For example, we speculate that the two species’ different foraging strategies may explain our results: the genetic component may be more influential in affecting individual foraging performance in *M*. *arenarius*. Differently, the genetic component may be more influential in affecting aggression in the group forager, *M*. sp., an inherently more aggressive species. In addition, we predicted that the specific aggression level would increase with phylogenetic distance and would be the highest against heterospecifics, followed by conspecifics, and the lowest against nestmates. Both species confirmed this prediction and demonstrated higher aggression towards heterospecifics and lower aggression towards conspecifics and nestmates. Lastly, *M*. *arenarius* spent less time interacting with heterospecifics compared to conspecifics and nestmates, while *M*. sp. showed a tendency towards the opposite pattern.

### The correlation between relatedness and colony performance

Many studies have reported the positive effects of colony diversity on foraging in honeybees^20,73–75^ (the more diverse, the more successful). In ants, colony genetic diversity effects have been suggested in only a few species, mostly those featuring highly polyandrous queens^19,27,30,31^, but see^26^, which was not the case here. In the present study, foraging performance positively correlated with genetic diversity in *M*. *arenarius* but not in *M*. sp. These contrasting results could perhaps be explained by the different foraging strategies of the two species. Individually-foraging species are probably more affected by genetic variance within colonies, because each worker makes decisions independently. In contrast, group foragers recruit and forage on a trail and are heavily influenced by pheromones that could mask differences in individual behavior. Supporting our suggestion, other studies that have focused on species that forage either individually or sometimes individually, have also demonstrated a positive correlation between inter-worker genetic variance and behavioral diversity^19,27^. Similarly, phenotypic divergence contributes to fitness in social arthropods that forage individually^24,76^. Alternatively, the different link found between genetic diversity and performance in the two species, might be explained by their different mating systems (see the discussion of “Genetic architecture of colonies” below). Importantly, we note that the interspecific differences probably derive from many traits. A rigorous way by which to test these suggestions would be to employ the comparative method and study either related species or populations of the same species that differ in their foraging strategy and to quantify their within-colony genetic relatedness. In short, only the application of many interspecific comparisons will be able to assess the generality of an evolutionary phenomenon.

We are not familiar with any study demonstrating how genetic diversity affects nest maintenance, other than a study that found a positive effect of genetic diversity on the efficiency of colony thermoregulation in honeybees^28,29^. A few studies have suggested the positive contribution of genetic diversity to nest construction^26,27^, but its ongoing maintenance is more frequently performed, and no less important. The nest entrance is the deposition site of colony-specific pheromones, enabling unrestricted access in and out of the colony, and it is the location where interactions with potential invaders usually take place^77–80^. A positive link between colony genetic diversity and nest maintenance was found here only for *M*. *arenarius*, similar to its link with foraging performance. The reason for this could be the association between foraging and nest maintenance behavior. After the nest entrance in another harvester ant species (*Pogonomyrmex barbatus*) was blocked, foragers encountered difficulty in leaving the nest, leading to a switch of tasks from foraging to nest maintenance^52,53^. This suggests that the foragers are also those involved in nest maintenance, and that more efficient foraging implies more efficient nest maintenance.

Our field experiments experienced several limitations. First, we ignored the possible effects of phenotypic plasticity. Hasegawa *et al*.^81^, for instance, showed that genetically cloned ant workers vary in their phenotype, expressed as sucrose response threshold. This might be explained by learning, developmental fluctuations, or epigenetics. Since we collected the colonies randomly, we assumed that the colonies represented the “average value” of the common phenotype. However, the best way to test our first goal (e.g., the prediction that within-colony genetic diversity would be positively correlated with foraging and nest maintenance performances) could have been by controlled laboratory experiments using an ant species that produces genetic clones. We could thereby have separated between the genetic and phenotypic components and their effect on behavior. Second, colony size was unknown. In the studied species, excavating complete colonies and rearing them under laboratory conditions has proven to be almost impossible. Indeed, all past studies involving *M*. *arenarius* and similar *Messor* species were also performed in the field^47–49,51,72,82–89^. One method by which to estimate colony size in field studies is the mark-release-recapture method^90–92^. However, if only a small proportion of colony workers are outdoor-foragers, and most of the population resides underground, the ‘equal catchability’ assumption of this method^93^ cannot be applied to ants. Consequently, we estimated colony size according to the number of foragers (10–14% of the total colony size, as demonstrated in past studies). According to our findings, the two species differed in their colony size, which could have affected performance. Larger colonies could have foraged more intensively and removed more obstacles compared to smaller ones. In the two studied species, colony size overlaps with foraging strategy; the group forager has larger colonies than the individual forager (also reviewed in^71^). This could offer an alternative explanation for the obtained differences between the species, at least in the foraging and nest maintenance tasks.

### Aggression and interaction assays

We found that genetic diversity positively correlated with aggression level, in contrast to our prediction. Low genetic diversity could lead to the absence of cues on the cuticle or to a high resemblance among the profiles of different colonies that make separate colonies resemble as a single large colony, and thereby result in low aggression against non-nestmates^94^. Indirect evidence for this comes from studies of invasive ant species: many such species demonstrate low genetic diversity and very low aggression among colonies, leading to the creation of “super-colonies”^95^. Specifically, genetic diversity was positively correlated here, with the overall aggression level in *M*. sp. but not in *M*. *arenarius*. As a group forager, *M*. sp. is inherently aggressive due to its recruitment behavior. Therefore, the genetic component might have a stronger impact in this species and perhaps this is why we found this correlation in the group forager but not in the individual forager. Although the individual forager *M*. *arenarius* is larger^51^ and quite abundant^48^, it might lose in direct conflicts with group-foraging ants that can more successfully exploit and dominate food resources. Similarly, in a parallel system, the group forager *M*. *ebeninus* better exploits and dominates food patches compared to *M*. *arenarius*^47^. Similar to our finding for *M*. *arenarius*, other studies too did not detect any correlation between genetic diversity and aggression^34,96^.

Aggression level increased from nestmates and conspecifics to heterospecifics in both species. One mechanism that may explain this is that of a gradually more aggressive response to odor or template with increasing dissimilarity^97–99^. Although odor effects were not tested here, we speculate that aggression levels might have been derived from odor-based recognition cues, as commonly seen in ant research. From an ecological point of view, aggression should be higher towards conspecifics and not heterospecifics, as the former are perceived as the ultimate competitors. However, in these two harvester ant species that presumably share their ecological niche, at least in regard to resources collected and foraging territory^72^ (Pers. Obs., M. S.), it is reasonable to assume that higher aggression would be directed towards heterospecifics. In support of this, in addition to being more aggressive towards heterospecifics, it seems that in *M*. sp. the correlation between relatedness and overall aggression level was based mainly on the aggression towards heterospecifics (see Figure S3 in the Supplementary Material). Aggressive responses were similar in both species, but the interaction pattern differed between them. Co-occurring species do not necessarily demonstrate a symmetrical response to one another. For example, in conflicts over food between two sympatric gerbil species, one is more aggressive than the other^100^. Even in a system of two co-occurring ant species that sometimes share a single nest, one species can be more aggressive to the other^101^.

The different interaction levels between the two species could have resulted from a behavior observed only in *M*. sp.: in the aggression assays, workers clung to the legs and other body parts of the larger *M*. *arenarius*. We interpret this behavior as the highest level of aggression, reflected in prolonged interaction time. This suggests that *M*. sp. directly attacks heterospecifics, while *M*. *arenarius* often avoids heterospecifics (also implied in^48^), reflected in a shorter interaction time. We observed this behavior in the field as well. These two species frequently interact in the field, as they have overlapping foraging territories (Pers. Obs., M. S.). Consequently, although the two species differ in body size, we do not believe that size had an impact on their tendency to interact. In general, body size may affect the collection of different load sizes of food items^102,103^; although another study in Israel compared between *M*. *arenarius* and *M*. *ebeninus*, a species similar in size to *M*. sp., and found that they collect similar load sizes^72^.

Surprisingly, we did not find a correlation between the number of patrilines in *M*. *arenarius* and colony performance or aggression level. This was despite our expectation that, in a monogynous and polyandrous mating system, the number of queen mates would directly affect the genetic diversity among workers (the more patrilines – the more diverse workers). It is nevertheless possible that there is a skew in paternity, meaning that some drones sire more offspring than others. Perhaps such a skew is more influential than the number of patrilines in the colony. In some species of honeybees, for instance, siring success is biased towards the first or last drones mating with the queen^104,105^ but see^106^, and thus the actual number of the queen’s mates matters less. Nonetheless, we still found a trend of a positive correlation between patriline number and nest maintenance in *M*. *arenarius*, in line with the positive correlation between genetic diversity and nest maintenance in this species.

### Genetic architecture of colonies

Queen polyandry, found only in *M*. *arenarius*, is a rather common feature in ants and has evolved in 13 genera^107^. It appears adaptive but nonetheless costly. For instance, large sperm storage reduces immunity against pathogens during colony founding^108^ and increases potential disease transmission from drones to queens^109,110^. However, this potential cost could be outweighed by the benefit of multiple patrilines that generate a specialized worker force in the colonies^13^. Regarding the occurrence of more than a single queen in *M*. sp. (polygyny), the benefits and costs of increasing genetic diversity probably hold true here as well, and are possibly even more extreme than in polyandrous colonies, due to the elevated genetic diversity. As noted, multiple mating is costly^111^, and therefore either polygyny or polyandry is sufficient to gain the benefits of diversity in ants (but see^112^). In support of this, the rate of polyandry has been shown to be lower in polygynous ant species than in monogynous ones^113^.

The distinct mating systems of the two species might also have contributed to the differences found in the link between genetic diversity and performance (i.e., foraging and nest maintenance performance). The mean relatedness index is higher in *M*. *arenarius* compared to *M*. sp.; thus, *M*. sp. colonies are more diverse than *M*. *arenarius* colonies. The link between genetic diversity and performance might not be strictly linear. At lower levels of diversity (such as in the case of *M*. *arenarius*), performance may be linearly linked, and the effect of diversity on performance is detectable. However, at higher levels of diversity (such as in the case of *M*. sp.), it may level-off, making the effect of diversity on performance harder to detect.

In summary, the co-occurring *M*. *arenarius* and *M*. sp. differ in their foraging strategy, colony size, body size, and mating system. We detected inter-specific differences in the link between within-colony genetic diversity and the foraging and nest maintenance performances, and aggression level. As a short take-home message, we suggest that the different foraging strategies may explain our results: innate genetic structure might have a higher impact on performance in an individually decision-making forager, such as *M*. *arenarius*; whereas innate genetic structure may exert a greater influence on aggressive behavior in a group forager, such as *M*. sp. Finally, the different mating systems may explain our findings, but only regarding foraging and nest maintenance performances, not regarding aggression level. It could be that genetic diversity is more linked to these performances of *M*. *arenarius*, because this species is generally less diverse than *M*. sp.

## Electronic supplementary material

Supplementary Information

Dataset
