# Supplementary material for: Within-colony genetic diversity differentially affects foraging, nest maintenance, and aggression in two species of harvester ants
Source: Sci Rep. 2018 Sep 14;8:13868. doi: 10.1038/s41598-018-32064-3 (PMC6138738; doi:10.1038/s41598-018-32064-3)
Supplement: Supplementary file 1 — Supplementary Information [file 41598_2018_32064_MOESM1_ESM.pdf]

Supplementary Material for the manuscript

Within-colony genetic diversity differentially affects foraging, nest maintenance, and aggression in two species of harvester ants

MAYA SAAR, PIERRE-ANDRÉ EYER, TAL KILON-KALLNER, ABRAHAM HEFETZ& INON SCHARF

The Supplementary Material consists of five tables, a short description of the molecular analysis conducted, and four figures.

Table S1: Characters for recognizing and distinguishing between *Messor arenarius* and *Messor* sp.:

**Character**	***Messor arenarius***	***Messor* sp.**
Max. total length (TL)	16 mm	8.3 mm
Funiculus segments	1st segment slightly longer and wider than 2nd	1st segment distinctly longer and wider than the 2nd, somewhat flattened dorso-ventrally
Propodeum	posteriorly armed, with acute spine	unarmed, posteriorly angular
Petiole node	truncated, anterior and truncated surfaces meet at right to obtuse angle.(in small specimens, occasionally rounded dorsally)	never truncated, anterior and posterior surfaces meet at sharp, acute angle (< 45°)
Colouration and texture	head dorsum matte, strongly striate punctuate, anterior surface of petiole and of postpetiole, and gastertergites matte, densely punctuated	head, anterior surface of petiole and postpetiole, and gaster shiny; head finely striate longitudinally with sparse points; in largest specimensocciput with transversal striation
Setation of 1^{st}gaster segment	numerous (> 80) uniformly distributed, long, decumbent to erect setae; setae length up to 0.35–0.40 mm	few (< 40), short setae concentrated on antero-dorsal surface of tergite; distinctly shorter (< 0.15 mm) than row of setae on posterior margin of tergite (0.20–0.25 mm)

Table S2: The foraging and nest maintenance data, obtained in two consecutive days (9 and 18 colonies of *M*. sp. and *M. arenarius* respectively). The mean of data obtained in the two days was tested in correlation with the genetic relatedness (queller index). The molecular analysis data is also presented (9 and 15 colonies of *M*. sp. and *M. arenarius* respectively):

Species / Colony ID	D1 mean seed taken	D2 mean seed taken	Seed taken (mean D1, 2)	Dist from Het colony (m)	Dist from Con colony (m)	D1 tp place	D1 tp rem	D2 tp place	D2 tp rem	D1 prop	D2 prop	Prop (mean D1, D2)	No. Worker genotyped	Queller index	Patriline no.
***Messor* sp.**															
2	0.05	0.13	0.09	7.50	4.60	33	2	28	0	0.06	0.00	0.03	11	0.07	
3	0.01	0.01	0.01	29.40		5	0	5	0	0.00	0.00	0.00	9	0.32	
5	0.02	0.02	0.02	28	50.50	5	0	6	0	0.00	0.00	0.00	12	0.18	
6	0.06	1.00	0.53	14.40	30.30	15	0			0.00		0.00	8	0.45	
7	0.20	1.02	0.61	27.70	58	16	7	21	10	0.44	0.48	0.46	12	0.22	
8	0.26	0.83	0.55	34.70	50.10	8	0	8	1	0.00	0.13	0.06	12	0.33	
14	0.14	0.79	0.47	55	58	25	0	16	3	0.00	0.19	0.09	12	0.13	
40	0.02	0.02	0.02	46.10	1.60	9	2	9	2	0.22	0.22	0.22	12	0.15	
847	0.58	0.05	0.32	18.20	50.10	6	0	6	3	0.00	0.50	0.25	15	0.19	
Messor arenarius															
6	0.68	0.86	0.77	28.50	80.50	14	4	14	2	0.29	0.14	0.21	12	0.31	3
18	2.01		2.01			18	6	17	16	0.33	0.94	0.64			
19	0.31		0.31			12	11	12	9	0.92	0.75	0.83			
20	0.13		0.13			8	3	8	4	0.38	0.50	0.44			

21	0.97	0.29	0.63	9.30	38	10	2	10	0	0.20	0.00	0.10	12	0.89	1
22	0.03	0.02	0.02			12	0	12	3	0.00	0.25	0.13			
23	0.02	0.03	0.02	46.10	12	9	2	11	6	0.22	0.55	0.38	21	0.44	3
24	1.51	1.93	1.72	1.60	4.80	13	2	17	3	0.15	0.18	0.17	12	0.37	2
25	1.37	1.59	1.48	1.72	7.90	14	2	14	13	0.14	0.93	0.54	11	0.27	3
26	0.03	0.06	0.05	18.10	60	6	0	6	0	0.00	0.00	0.00	12	0.81	1
27	0.03	0.02	0.03	16.10	9.10	11	0	11	1	0.00	0.09	0.05	12	0.63	2
28	0.05	0.28	0.16	20.50	128	13	5	14	5	0.38	0.36	0.37	11	0.56	2
30	0.73	0.76	0.74			12	5	12	2	0.42	0.17	0.29	12	0.48	1
31	0.94	1.81	1.38	115	112	16	5	16	4	0.31	0.25	0.28	12	0.18	3
33	0.63	1.85	1.24	9	14.40	11	5	11	4	0.45	0.36	0.41	12	0.28	3
34													11		1
210	0.05	0.03	0.04			9	2	12	3	0.22	0.25	0.24	12	0.67	1
852	0.03	0.19	0.11	2.10	80.50	9	1	9	1	0.11	0.11	0.11	11	0.70	2
861	0.06	0.15	0.11	12.70	28.40	10	0	10	0	0.00	0.00	0.00	10	0.56	2

Abbreviations in headlines for Table S2: D = Day, Dist = Distance, Het = heterospecific, Con = conspecific, tp = toothpicks, place = placed, rem = removed, prop = proportion and Queller index = Queller relatedness index.

Table S3: The aggression assay data for 9 and 12 colonies of *M*. sp. and *M. arenarius*, respectively. Specific aggression levels (towards conspecifics, or heterospecifics, or nestmates) were averaged per colony:

Species /Colony ID	No. workers in Con	Aggression Con (mean)	No. workers in Het	Aggression Het (mean)	No. workers in Nestmate	Aggression Nestmates (mean)	Sum of aggression
***Messor* sp.**							
2	12	2.49	8	3.88	8	0.00	6.37
3		0.00	9	2.12	7	0.02	2.14
5	10	0.13	10	2.54	9	0.33	3.00
6	5	0.11	5	0.71	5	0.04	0.86
7	12	0.65	12	5.09	8	0.00	5.74
8	12	0.69	6	2.11	8	0.61	3.41
14	12	0.36	12	5.98	8	0.00	6.34
40	10	0.71	11	2.91	8	0.16	3.78
847	11	0.32	11	2.62	8	0.51	3.45
Messor arenarius							
6	5	0.17	11	2.55	8	0.24	2.96
18							
19							

20							
21	12	0.78	11	4.47	6	0.21	5.46
22							
23	0	0.00	11	1.53	8	0.06	1.60
24	12	0.47	8	1.56	3	0.01	2.03
25	6	0.48	6	1.44	4	0.09	2.01
26	8	0.13	4	4.24	5	0.00	4.37
27	6	0.31	6	1.10	4	0.00	1.41
28	12	0.69	12	2.84	8	0.01	3.55
30							
31	12	0.75	12	3.98	8	0.10	4.82
33							
34							
210	10	0.07	9	1.83	8	0.08	1.98
852	11	0.07	11	1.64	8	0.10	1.81
861	12	0.40	11	4.97	7	0.05	5.42

Abbreviations in headlines for Table S3: Het = heterospecific encounter,

Con = conspecific encounter and Nestmate = nestmates encounter.

Table S4: Newly-developed PCR conditions for the microsatellite markers to fit and amplify the alleles of the two studies *Messor* species:

Species and markers	Set name and volume	Annealing temperature (°C)
***M.*sp**		
	**Sp1**	62
Ms2D	0.3 µl	
Asen155	0.4 µl	
	**Sp2**	54
Asen15	0.4 µl	
	**Sp3**	52
Ms1F	0.3µl	
Ms1B	0.6 µl	
M. arenarius		
	**arenarius1**	62
Ms2D	0.3 µl	
Asen155	0.4 µl	
	**arenarius2**	57
Ms1A	0.3 µl	
Ms2C	0.5 µl	
Ms1F	0.5 µl	
	**arenarius3**	52
Asen15	0.3 µl	

Table S5: The genotyping data of 9 and 15 colonies of *M.* sp. and *M. arenarius*, respectively. Individuals (for instance, on the first row; Msp847w1) are labeled as follows: species, colony ID and the worker number. For each individual, the number of basepairs is indicated for the two alleles of every microsatellite marker analyzed:

**Species**	**Microsatellite markers**													
***Messor* sp.**	v15		1F		2D		m155		1B		1A		2C	
Msp847w1	x	x	x	x	204	210	190	192	x	x	NA	NA	NA	NA
Msp847w2	x	x	x	x	210	212	192	192	x	x	NA	NA	NA	NA
Msp847w3	132	155	163	177	210	220	188	192	175	175	NA	NA	NA	NA
Msp847w163	132	155	169	177	210	212	188	192	175	175	NA	NA	NA	NA
Msp847w164	132	136	169	177	210	212	192	192	175	175	NA	NA	NA	NA
Msp847w165	132	155	169	177	210	220	188	192	175	175	NA	NA	NA	NA
Msp847w166	136	159	163	169	210	226	192	194	175	177	NA	NA	NA	NA
Msp847w167	132	163	165	187	202	210	190	192	175	177	NA	NA	NA	NA
Msp847w168	147	171	169	189	202	212	192	205	175	177	NA	NA	NA	NA
Msp847w169	147	159	165	167	200	212	192	192	177	177	NA	NA	NA	NA
Msp847w170	132	136	169	177	210	220	188	192	175	177	NA	NA	NA	NA
Msp847w171	132	155	169	177	210	220	192	192	175	175	NA	NA	NA	NA
Msp847w172	157	157	163	169	202	212	192	194	177	177	NA	NA	NA	NA
Msp847w173	132	155	169	177	210	220	188	192	175	177	NA	NA	NA	NA
Msp847w174	157	163	163	165	202	212	192	194	175	177	NA	NA	NA	NA
Msp7w090	136	149	169	169	202	210	190	205	175	177	NA	NA	NA	NA
Msp7w091	147	163	169	169	200	204	190	202	175	175	NA	NA	NA	NA
Msp7w092	136	149	169	169	202	210	190	205	175	177	NA	NA	NA	NA

Msp7w093	132	165	161	167	210	228	190	196	175	177	NA	NA	NA	NA
Msp7w094	149	163	169	169	202	204	192	205	175	177	NA	NA	NA	NA
Msp7w095	149	163	169	169	202	210	190	205	175	177	NA	NA	NA	NA
Msp7w124	149	163	169	169	202	204	190	192	175	177	NA	NA	NA	NA
Msp7w125	163	165	165	169	202	210	190	215	175	177	NA	NA	NA	NA
Msp7w126	163	165	165	165	202	202	190	215	175	177	NA	NA	NA	NA
Msp7w127	147	163	169	169	200	210	190	192	175	175	NA	NA	NA	NA
Msp7w128	x	x	161	165	x	x	x	x	175	177	NA	NA	NA	NA
Msp7w129	136	149	x	x	202	204	190	205	x	x	NA	NA	NA	NA
Msp2w34	149	157	169	177	202	232	190	194	x	x	NA	NA	NA	NA
Msp2w35	157	171	169	173	204	210	190	194	x	x	NA	NA	NA	NA
Msp2w36	136	149	167	175	204	210	188	192	175	175	NA	NA	NA	NA
Msp2w37	136	171	167	175	202	210	188	215	175	177	NA	NA	NA	NA
Msp2w38	136	171	167	187	202	210	188	205	175	175	NA	NA	NA	NA
Msp2w55	143	157	169	171	210	226	188	192	175	177	NA	NA	NA	NA
Msp2w56	171	171	165	175	202	204	192	215	177	177	NA	NA	NA	NA
Msp2w57	143	157	165	169	210	226	190	192	175	177	NA	NA	NA	NA
Msp2w58	143	157	165	169	210	226	188	192	175	177	NA	NA	NA	NA
Msp2w59	143	157	169	171	210	226	188	192	175	177	NA	NA	NA	NA
Msp2w60	136	159	169	169	200	210	190	202	175	175	NA	NA	NA	NA
Msp14w131	157	163	161	165	204	226	192	202	177	177	NA	NA	NA	NA
Msp14w132	155	171	167	169	202	210	190	215	175	177	NA	NA	NA	NA
Msp14w133	157	163	161	165	204	214	192	202	177	177	NA	NA	NA	NA
Msp14w134	155	171	167	169	202	202	192	205	175	177	NA	NA	NA	NA
Msp14w135	157	171	167	169	202	210	190	190	175	177	NA	NA	NA	NA

Msp14w136	157	171	169	185	202	210	192	205	175	177	NA	NA	NA	NA
Msp14w137	157	163	161	165	204	214	192	194	177	177	NA	NA	NA	NA
Msp14w138	136	149	167	169	202	202	192	205	175	177	NA	NA	NA	NA
Msp14w139	157	163	161	165	204	214	190	194	177	177	NA	NA	NA	NA
Msp14w140	157	171	167	169	202	210	192	215	x	x	NA	NA	NA	NA
Msp14w141	155	171	167	169	202	202	192	205	175	177	NA	NA	NA	NA
Msp14w142	155	171	167	169	202	210	192	215	x	x	NA	NA	NA	NA
Msp40w211	136	149	163	169	204	204	192	202	177	177	NA	NA	NA	NA
Msp40w212	132	157	167	189	200	200	194	205	177	177	NA	NA	NA	NA
Msp40w213	136	163	163	165	204	210	192	205	175	177	NA	NA	NA	NA
Msp40w214	136	149	163	169	204	204	192	205	177	177	NA	NA	NA	NA
Msp40w215	147	149	169	169	210	218	190	194	175	177	NA	NA	NA	NA
Msp40w216	136	149	163	169	204	204	192	192	177	177	NA	NA	NA	NA
Msp40w217	149	163	169	169	210	218	190	194	175	175	NA	NA	NA	NA
Msp40w218	157	163	167	189	200	200	194	209	177	177	NA	NA	NA	NA
Msp40w219	136	149	163	169	204	204	192	192	177	177	NA	NA	NA	NA
Msp40w220	132	157	187	189	200	210	194	205	177	177	NA	NA	NA	NA
Msp40w221	136	149	163	169	204	204	192	205	177	195	NA	NA	NA	NA
Msp40w222	132	157	167	189	200	200	194	209	177	177	NA	NA	NA	NA
Msp8w181	151	159	167	173	204	204	192	192	175	177	NA	NA	NA	NA
Msp8w182	149	159	167	169	204	204	192	196	175	177	NA	NA	NA	NA
Msp8w183	149	159	167	173	204	204	192	192	175	177	NA	NA	NA	NA
Msp8w184	149	159	167	169	202	204	192	192	175	177	NA	NA	NA	NA
Msp8w185	149	159	167	173	202	204	192	192	175	177	NA	NA	NA	NA
Msp8w186	149	159	167	169	202	204	192	192	175	177	NA	NA	NA	NA

Msp8w187	136	163	165	167	202	226	192	194	177	177	NA	NA	NA	NA
Msp8w188	149	159	167	173	204	204	192	192	175	177	NA	NA	NA	NA
Msp8w189	155	157	167	169	202	210	192	194	175	177	NA	NA	NA	NA
Msp8w190	151	159	167	173	202	204	192	196	175	175	NA	NA	NA	NA
Msp8w191	136	163	165	167	226	226	192	194	175	177	NA	NA	NA	NA
Msp8w192	136	163	165	167	202	226	192	194	175	177	NA	NA	NA	NA
Msp3w246	143	143	160	169	204	204	192	202	175	175	NA	NA	NA	NA
Msp3w247	161	161	165	175	204	214	192	215	175	175	NA	NA	NA	NA
Msp3w248	143	143	160	169	204	204	192	202	175	175	NA	NA	NA	NA
Msp3w252	143	143	160	169	204	204	192	202	x	x	NA	NA	NA	NA
Msp3w253	143	157	167	169	204	204	192	194	x	x	NA	NA	NA	NA
Msp3w254	143	143	160	169	204	204	192	202	x	x	NA	NA	NA	NA
Msp3w255	143	157	160	169	204	204	190	192	x	x	NA	NA	NA	NA
Msp3w256	157	159	167	175	202	202	190	192	x	x	NA	NA	NA	NA
Msp3w257	153	157	169	175	202	202	190	192	x	x	NA	NA	NA	NA
Msp5w234	153	163	167	167	204	210	192	200	x	x	NA	NA	NA	NA
Msp5w235	140	171	165	169	202	212	190	192	x	x	NA	NA	NA	NA
Msp5w236	140	171	163	167	202	210	190	192	x	x	NA	NA	NA	NA
Msp5w237	136	163	165	169	202	204	192	202	x	x	NA	NA	NA	NA
Msp5w238	140	149	163	163	202	212	190	192	x	x	NA	NA	NA	NA
Msp5w239	136	163	163	169	202	204	192	192	x	x	NA	NA	NA	NA
Msp5w240	140	149	163	163	202	210	190	192	x	x	NA	NA	NA	NA
Msp5w241	140	171	163	163	202	212	190	192	x	x	NA	NA	NA	NA
Msp5w242	140	149	163	167	202	212	190	192	x	x	NA	NA	NA	NA
Msp5w243	153	163	167	171	204	210	192	221	x	x	NA	NA	NA	NA

Msp5w244	140	171	165	169	202	210	190	192	x	x	NA	NA	NA	NA
Msp5w245	149	171	167	167	204	210	190	192	x	x	NA	NA	NA	NA
Msp6w264	145	163	165	167	210	224	190	205	x	x	NA	NA	NA	NA
Msp6w265	145	163	167	169	210	224	190	205	x	x	NA	NA	NA	NA
Msp6w266	145	163	165	167	204	210	190	205	x	x	NA	NA	NA	NA
Msp6w267	145	163	165	167	204	210	190	205	x	x	NA	NA	NA	NA
Msp6w268-R	157	157	165	167	202	202	190	192	x	x	NA	NA	NA	NA
Msp6w269	143	163	165	167	204	210	190	205	x	x	NA	NA	NA	NA
Msp6w270-R	147	157	165	165	210	212	190	192	x	x	NA	NA	NA	NA
Msp6w271	145	163	165	167	210	224	190	205	x	x	NA	NA	NA	NA
Messor arenarius														
Ma21in58	128	128	179	179	206	214	177	177	NA	NA	162	164	168	168
Ma21in59	128	128	179	179	206	214	177	177	NA	NA	162	164	168	168
Ma21in60	128	128	179	179	198	214	177	177	NA	NA	162	182	168	168
Ma21in66	128	128	179	179	198	214	177	193	NA	NA	162	164	168	168
Ma21in67	128	128	179	179	206	214	177	193	NA	NA	162	164	168	168
Ma21in68	128	128	179	179	198	214	177	193	NA	NA	162	182	168	168
Ma21f534	128	128	179	179	206	214	177	177	NA	NA	162	164	168	168
Ma21f535	128	128	179	179	198	214	177	177	NA	NA	162	164	168	168
Ma21f536	128	128	179	179	206	214	177	177	NA	NA	162	182	168	168
Ma21f537	128	128	179	179	206	214	177	193	NA	NA	162	164	168	168
Ma21f538	128	128	179	179	206	214	177	193	NA	NA	162	182	168	174
Ma21f539	128	128	179	179	198	214	177	193	NA	NA	162	164	168	174
Ma861w2	118	118	173	182	210	212	x	x	NA	NA	162	182	174	178
Ma861w3	118	118	173	182	200	214	x	x	NA	NA	182	184	178	178

Ma861w4	118	118	175	182	190	214	x	x	NA	NA	x	x	x	x
Ma861w5	118	118	175	182	190	214	x	x	NA	NA	x	x	x	x
Ma861w6	115	115	173	177	200	214	x	x	NA	NA	x	x	x	x
Ma861w7	115	115	173	182	214	214	x	x	NA	NA	182	184	174	178
Ma861w8	118	118	175	182	190	214	x	x	NA	NA	x	x		
Ma861w10	118	118	173	182	200	214	x	x	NA	NA	x	x	x	x
Ma861w11	118	118	173	177	214	214	x	x	NA	NA	x	x	x	x
Ma861w12	115	115	173	177	200	214	x	x	NA	NA	x	x	x	x
Ma6s159	x	x	173	182	214	214	177	193	NA	NA	162	182	178	178
Ma6s160	x	x	175	177	190	210	177	193	NA	NA	162	162	168	178
Ma6s161	x	x	175	182	190	214	177	193	NA	NA	162	184	168	178
Ma6s179	115	115	173	177	200	214	177	181	NA	NA	162	182	178	178
Ma6s180	119	119	173	182	214	214	177	181	NA	NA	162	182	178	178
Ma6s181	119	119	173	177	200	214	177	181	NA	NA	182	184	178	178
Ma6f580	115	115	173	177	214	214	177	181	NA	NA	162	182	178	178
Ma6f581	115	115	175	182	190	214	177	177	NA	NA	162	162	168	174
Ma6f582	115	115	173	177	214	214	177	181	NA	NA	162	182	178	178
Ma6w597	119	119	175	182	190	214	x	x	NA	NA	162	184	168	174
Ma6w598	115	115	175	182	190	190	x	x	NA	NA	162	184	168	178
Ma6w599	119	119	175	182	190	214	x	x	NA	NA	162	184	168	174
Ma24w1	115	115	173	177	214	214	x	x	NA	NA	182	184	178	178
Ma24w2	119	119	175	182	190	214	x	x	NA	NA	162	162	168	178
Ma24w3	119	119	173	177	214	214	x	x	NA	NA	162	182	178	178
Ma24w4	119	119	173	177	x	x	x	x	NA	NA	162	182	178	178
Ma24s192	119	119	173	177	214	214	177	181	NA	NA	182	184	174	178

Ma24s193	119	119	175	177	190	214	177	177	NA	NA	162	162	168	178
Ma24s194	115	115	173	182	214	214	177	181	NA	NA	162	182	174	178
Ma24s195	x	x	175	182	190	214	177	193	NA	NA	162	184	168	174
Ma24s196	x	x	175	182	190	214	177	193	NA	NA	162	184	168	178
Ma24s197	x	x	175	177	190	214	177	193	NA	NA	162	184	168	178
Maf529	115	115	175	177	190	214	177	177	NA	NA	162	162	168	178
Maf530	119	119	173	182	214	214	177	181	NA	NA	182	184	174	178
Ma25in22	x	x	175	177	190	214	177	193	NA	NA	162	162	168	178
Ma25in23	x	x	175	177	190	214	177	193	NA	NA	162	162	168	178
Ma25in24	x	x	175	177	190	214	177	193	NA	NA	162	162	168	178
Ma25in40	115	115	175	182	190	214	177	177	NA	NA	162	184	168	178
Ma25in41	115	115	173	177	200	214	177	181	NA	NA	182	184	174	178
Ma25in42	119	119	175	177	190	214	177	177	NA	NA	162	162	168	174
Ma25f531	119	119	173	182	200	214	177	181	NA	NA	182	184	174	178
Ma25f532	115	115	173	182	214	214	177	181	NA	NA	162	182	178	178
Ma25f540	x	x	175	177	190	214	x	x	NA	NA	162	162	168	178
Ma25f541	x	x	175	177	190	214	177	193	NA	NA	162	184	168	174
Ma25f542	x	x	173	182	x	x	177	193	NA	NA	182	184	178	178
Ma31w621	x	x	173	177	x	x	x	x	NA	NA	182	184	174	178
Ma31w622	x	x	175	177	190	214	x	x	NA	NA	162	184	168	174
Ma31w623	x	x	173	177	214	214	x	x	NA	NA	182	184	178	178
Ma31w624	x	x	175	177	190	190	x	x	NA	NA	162	162	168	x
Ma31w625	x	x	175	182	190	190	x	x	NA	NA	x	x	x	x
Ma31w626	x	x	175	182	190	214	x	x	NA	NA	162	184	168	174
Ma31w627	x	x	173	182	214	214	x	x	NA	NA	182	184	178	178

Ma31w628	x	x	173	182	214	214	x	x	NA	NA	162	184	178	178
Ma31w629	x	x	173	182	214	214	x	x	NA	NA	182	184	178	178
Ma31w630	x	x	175	177	190	214	x	x	NA	NA	162	162	168	178
Ma31w631	x	x	175	182	190	190	x	x	NA	NA	162	184	168	174
Ma31w632			173	182	210	214			NA	NA	182	184	174	178
Ma26in04	115	115	175	175	202	206	x	x	NA	NA	158	188	176	178
Ma26in05	115	115	175	182	202	206	177	177	NA	NA	158	188	174	178
Ma26in06	115	115	175	182	202	206	177	177	NA	NA	158	162	174	178
Ma26s141	x	x	175	175	202	206			NA	NA	x	x		
Ma26s142	x	x	175	182	202	206	177	193	NA	NA	158	162	176	178
Ma26s143	x	x	175	175	202	206	177	193	NA	NA	158	162	174	178
Ma26s144	115	115	175	175	202	206	177	177	NA	NA	158	162	x	x
Ma26s145	115	115	175	175	202	206	x	x	NA	NA	158	162	176	178
Ma26s146	115	115	175	182	202	206	177	177	NA	NA	158	162	174	178
Ma26f147	115	115	175	182	202	206	177	177	NA	NA	158	188	176	178
Ma26f148	x	x	175	175	202	206	x	x	NA	NA	x	x	x	x
Ma26f149	115	115	175	175	202	206	177	177	NA	NA	158	162	174	178
Ma27s84	115	115	175	182	210	210	177	195	NA	NA	162	166	x	x
Ma27s85	115	115	175	182	200	214	177	195	NA	NA	162	166	174	176
Ma27s86	115	115	175	182	210	210	177	195	NA	NA	162	166	174	176
Ma27s87	x	x	175	182	210	210	177	195	NA	NA	162	182	168	174
Ma27s88	x	x	175	175	200	214	177	195	NA	NA	162	166	174	176
Ma27s89	x	x	175	175	210	214	177	195	NA	NA	162	166	168	176
Ma27f156	115	115	175	175	210	214	177	195	NA	NA	162	166	174	168
Ma27f157	115	115	175	175	200	214	177	195	NA	NA	162	182	174	168

Ma27f158	115	115	175	175	210	210	177	195	NA	NA	162	166	174	176
Ma27F495	x	x	175	182	200	214	177	195	NA	NA	162	166	168	174
Ma27F496	x	x	175	182	200	212	177	193	NA	NA	162	166	168	174
Ma27F497	x	x	175	175	210	214	177	195	NA	NA	162	166	168	174
Ma852w570	x	x	175	175	202	204	x	x	NA	NA	162	186	174	176
Ma852w571	x	x	175	175	202	204	x	x	NA	NA	162	186	174	176
Ma852w572	x	x	175	175	202	202	x	x	NA	NA	162	186	174	176
Ma852w573	x	x	175	175	202	204	x	x	NA	NA	162	186	174	174
Ma852w574	x	x	175	175	202	202	x	x	NA	NA	162	186	174	174
Ma852w575	x	x	175	175	202	204	x	x	NA	NA	162	162	168	174
Ma852w576	x	x	175	179	202	202	x	x	NA	NA	184	186	176	176
Ma852w577	x	x	175	175	202	202	x	x	NA	NA	162	162	174	174
Ma852w578	x	x	175	175	202	202	x	x	NA	NA	162	186	174	174
Ma852w579	x	x	175	175	202	204	x	x	NA	NA	162	186	176	176
Ma852w601	x	x	175	175	202	202	x	x	NA	NA	162	186	174	174
Ma23w637	116	126	175	182	190	190	x	x	NA	NA	x	x	x	x
Ma23w638	116	126	173	182	190	210	x	x	NA	NA	x	x	x	x
Ma23w639	115	116	173	182	190	210	x	x	NA	NA	x	x	x	x
Ma23w640	116	126	173	182	190	210	x	x	NA	NA	x	x	x	x
Ma23w641	116	126	173	182	190	210	x	x	NA	NA	x	x	x	x
Ma23w642	115	115	175	175	190	202	x	x	NA	NA	x	x	x	x
Ma23w643	116	126	173	182	190	210	x	x	NA	NA	x	x	x	x
Ma23w644	116	126	173	182	190	210	x	x	NA	NA	x	x	x	x
Ma23w645	116	126	175	182	190	190	x	x	NA	NA	x	x	x	x
Ma23w646	118	126	175	182	190	190	x	x	NA	NA	162	186	174	174

Ma23w647	116	118	173	182	190	190	x	x	NA	NA	162	186	174	174
Ma23w648	126	126	173	175	190	202	x	x	NA	NA	162	186	174	176
Ma23w649	116	118	173	182	190	210	x	x	NA	NA	162	162	176	176
Ma23w650	116	118	173	182	190	210	x	x	NA	NA	162	162	176	176
Ma23w651	118	126	173	182	190	190	x	x	NA	NA	162	186	176	176
Ma23w652	126	126	173	173	202	210	x	x	NA	NA	186	186	176	176
Ma23w653	116	116	173	175	202	210	x	x	NA	NA	186	186	174	176
Ma23w654	116	116	175	175	190	202	x	x	NA	NA	186	186	174	176
Ma23w655	116	118	175	182	190	210	x	x	NA	NA	162	186	174	176
Ma23w656	118	126	175	182	190	190	x	x	NA	NA	162	186	174	176
Ma23w657	116	118	175	182	190	210	x	x	NA	NA	162	162	174	176
Ma28w658	116	116	182	182	204	204	x	x	NA	NA	162	182	174	178
Ma28w659	116	124	182	182	204	204	x	x	NA	NA	162	182	174	178
Ma28w660	124	126	175	182	210	210	x	x	NA	NA	162	164	178	178
Ma28w661	116	116	175	182	210	210	x	x	NA	NA	162	182	174	178
Ma28w662	116	124	182	182	210	210	x	x	NA	NA	162	182	174	178
Ma28w663	116	124	175	182	210	210	x	x	NA	NA	162	182	174	178
Ma28w664	116	124	175	182	204	204	x	x	NA	NA	162	182	174	178
Ma28w666	116	116	182	182	210	210	x	x	NA	NA	162	182	174	178
Ma28w667	116	124	182	182	210	210	x	x	NA	NA	162	182	174	178
Ma28w668	126	126	175	182	204	204	x	x	NA	NA	162	164	178	178
Ma28w669	116	116	182	182	210	210	x	x	NA	NA	162	182	174	178
Ma33w01	115	125	177	182	202	202	x	x	NA	NA	162	193	168	174
Ma33w02	115	116	179	179	202	202	x	x	NA	NA	162	167	168	168
Ma33w03	x	x	175	182	190	200	x	x	NA	NA	162	182	168	168

Ma33w04	115	115	175	177	200	200	x	x	NA	NA	162	182	168	168
Ma33w05	115	116	175	177	210	210	x	x	NA	NA	162	167	168	168
Ma33w06	115	116	175	177	210	210	x	x	NA	NA	162	167	168	168
Ma33w07	x	x	175	182	200	200	x	x	NA	NA	162	162	168	168
Ma33w08	115	115	175	182	210	210	x	x	NA	NA	x	x	x	x
Ma33w09	115	115	175	177	190	200	x	x	NA	NA	x	x	x	x
Ma33w10	115	115	177	179	210	210	x	x	NA	NA	x	x	x	x
Ma33w11	115	116	175	175	202	202	x	x	NA	NA	x	x	x	x
Ma33w12	115	115	175	177	200	200	x	x	NA	NA	x	x	x	x
Ma34w01	116	135	173	179	190	202	x	x	NA	NA	x	x	x	x
Ma34w02	115	115	175	179	190	202	x	x	NA	NA	x	x	x	x
Ma34w03	x	x	175	179	190	202	x	x	NA	NA	x	x	x	x
Ma34w04	x	x	x	x	190	202	x	x	NA	NA	x	x	x	x
Ma34w05	x	x	175	179	190	190	x	x	NA	NA	x	x	x	x
Ma34w07	115	115	175	179	190	202	x	x	NA	NA	x	x	x	x
Ma34w08	x	x	175	179	190	190	x	x	NA	NA	x	x	x	x
Ma34w09	115	115	175	179	190	190	x	x	NA	NA	x	x	x	x
Ma34w10	115	115	173	179	190	202	x	x	NA	NA	166	168	168	174
Ma34w11	115	115	175	179	190	202	x	x	NA	NA	166	168	168	174
Ma34w12	115	115	175	179	190	190	x	x	NA	NA	166	168	168	174
Ma210w679	x	x	173	179	190	202	x	x	NA	NA	166	182	168	178
Ma210w680	x	x	175	179	190	190	x	x	NA	NA	166	168	168	178
Ma210w681	x	x	173	179	190	190	x	x	NA	NA	166	168	168	174
Ma210w682	x	x	175	179	190	190	x	x	NA	NA	166	168	168	178
Ma210w683	x	x	175	179	190	190	x	x	NA	NA	166	168	168	178

Ma210w684	x	x	173	179	190	190	x	x	NA	NA	166	168	168	174
Ma210w685	x	x	175	179	190	190	x	x	NA	NA	166	168	168	174
Ma210w686	x	x	175	179	190	190	x	x	NA	NA	166	168	168	178
Ma210w687	x	x	175	179	190	202	x	x	NA	NA	166	168	168	174
Ma210w688	x	x	175	179	190	190	x	x	NA	NA	166	182	168	178
Ma210w689	x	x	173	179	190	190	x	x	NA	NA	166	168	168	174
Ma210w690	x	x	173	179	190	202	x	x	NA	NA	166	182	168	174
Ma30w1	116	135	175	179	190	190	x	x	NA	NA	166	168	168	178
Ma30w2	115	115	175	179	190	190	x	x	NA	NA	166	168	168	178
Ma30w3	115	115	175	179	190	202	x	x	NA	NA	x	x	x	x
Ma30w4	116	135	175	179	190	202	x	x	NA	NA	x	x	x	x
Ma30w5	116	135	173	179	190	202	x	x	NA	NA	x	x	x	x
Ma30w6	116	135	173	179	190	202	x	x	NA	NA	x	x	x	x
Ma30w7	115	115	173	179	190	202	x	x	NA	NA	x	x	x	x
Ma30w8	115	115	175	179	190	190	x	x	NA	NA	x	x	x	x
Ma30w9	115	115	173	179	190	190	x	x	NA	NA	x	x	x	x
Ma30w10	115	115	175	179	190	202	x	x	NA	NA	x	x	x	x
Ma30w11	115	115	175	179	190	190	x	x	NA	NA	x	x	x	x
Ma30w12	115	115	173	179	190	190	x	x	NA	NA	x	x	x	x

A short description of the molecular analysis conducted

For *M. sp*., five microsatellite markers were amplified. Polymerase chain reactions (hereafter, PCR) were carried out in three sets of co-amplified loci. For *M. arenarius*, six microsatellite markers were amplified. PCR were carried out in three sets of co-amplified loci (Table S4). For both species, the primer volumes required a 10µMol concentration and were equal for both *Forward* and *Reverse* primers. Each PCR was performed in a 15-µL volume containing 1 µL of DNA solution, 1µl of Q-Solution (Qiagen, excluding **arenarius2** set) and 7.5µl of 2X Taq-PCR Mastermix (Qiagen).Forward primers were 5'-end labeled with a fluorescent dye, either 6-FAM, HEX or TET. For each individual, the amplified products were post-PCR combined for genotyping analysis, in which alleles were separated on ABI 3730 capillary sequencer (Applied Biosystems, Foster City, CA, USA) and sized against Liz 600 sizing standards (BioVentures, Murfreesboro, TN, USA) using Gene Marker® HID (Holland & Parson 2011). Controls for genotyping errors due to null alleles were analyzed following the Expectation Maximization algorithm of Dempster et al. (1977) implemented in the FREE NA software (Chapuis&Estoup 2007). Estimation of linkage disequilibrium was performed in GENEPOP on the Web (Rousset 2008).

Figure S1: Laboratory aggression assays between *M. arenarius* (the large ant) and *M.*sp. (the small ant). Different levels of *M. arenarius* aggression towards *M.* sp.: (A) antennation, (B) mandibular threat, (C) short biting with the mandibles,(D) curling the guster and spraying. The threat run behavior is the only behavior missing here:

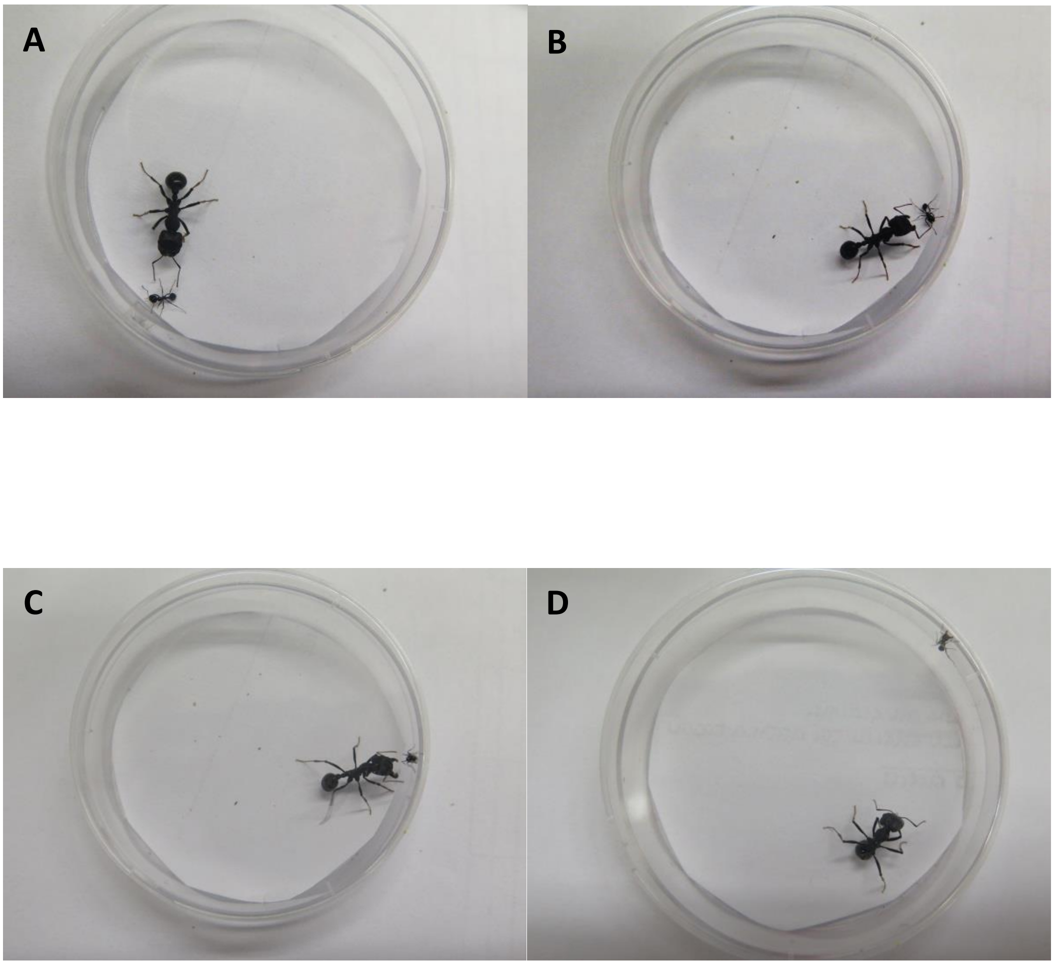

Figure S2: Foraging activities of the new species *M.*sp., observed in the study site; (A) a group of foragers ‘gang’ carrying a large food item back to their colony. (B) foragers collecting *Senecio joppensis* plant material at the end of their foraging trail. (C) measuring foraging trails in the study site using a measuring tape. (D) the colony entrance and foragers leaving and returning on the vegetation - clear foraging trail:

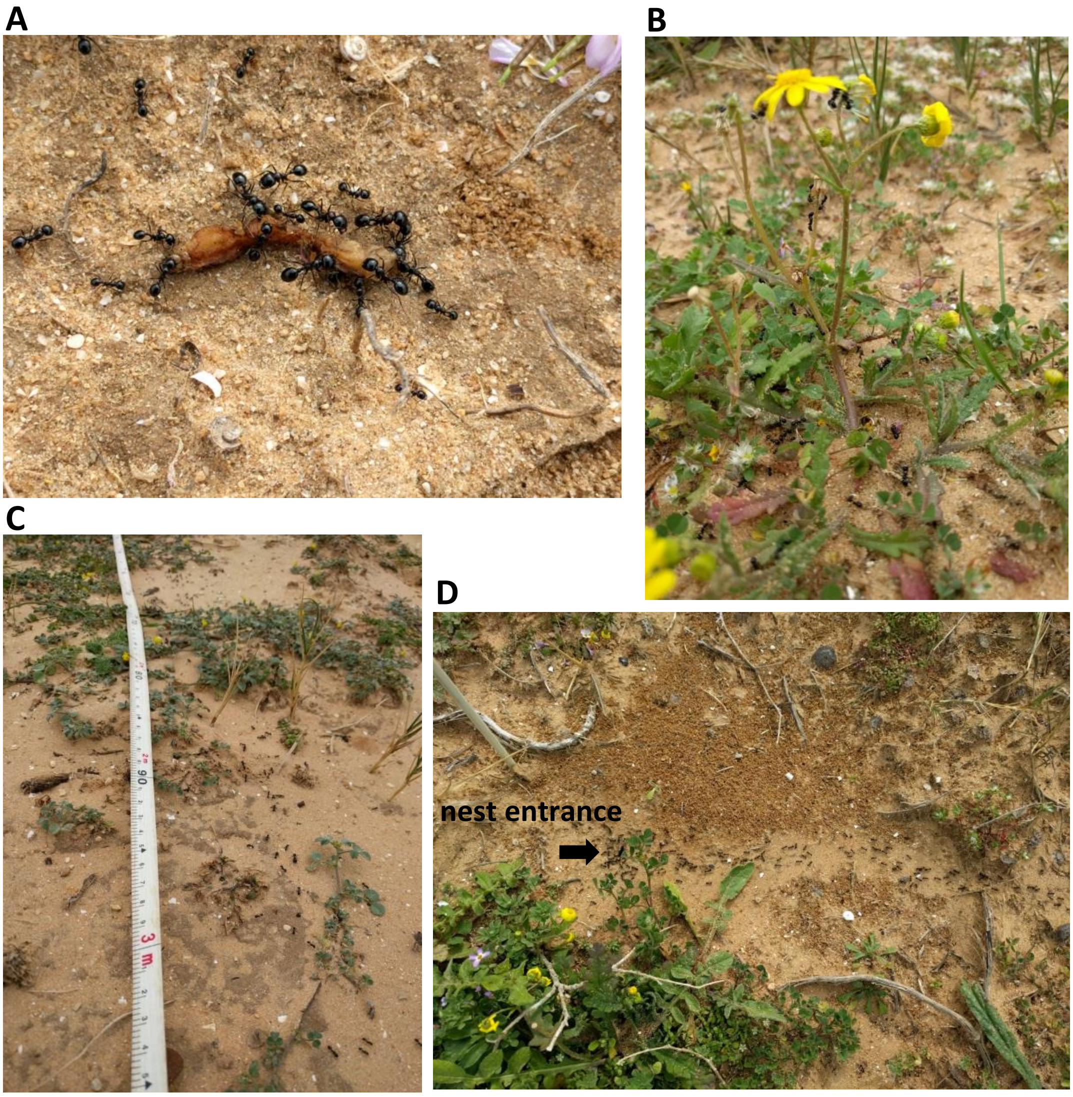

Figure S3: All correlations between genetic relatedness and the specific aggression levels in each studied species: Aggression towards conspecifics (A, D), towards heterospecifics (B, E) and towards nestmates (C, F). *M. arenarius'* correlations appear in grey dots (A-C) and *M.* sp.' correlations appear in black dots (D-F). The only significant correlation appears in (E): relatedness was negatively correlated with aggression towards heterospecifics in *M.* sp. All X axes present the Queller index for relatedness.

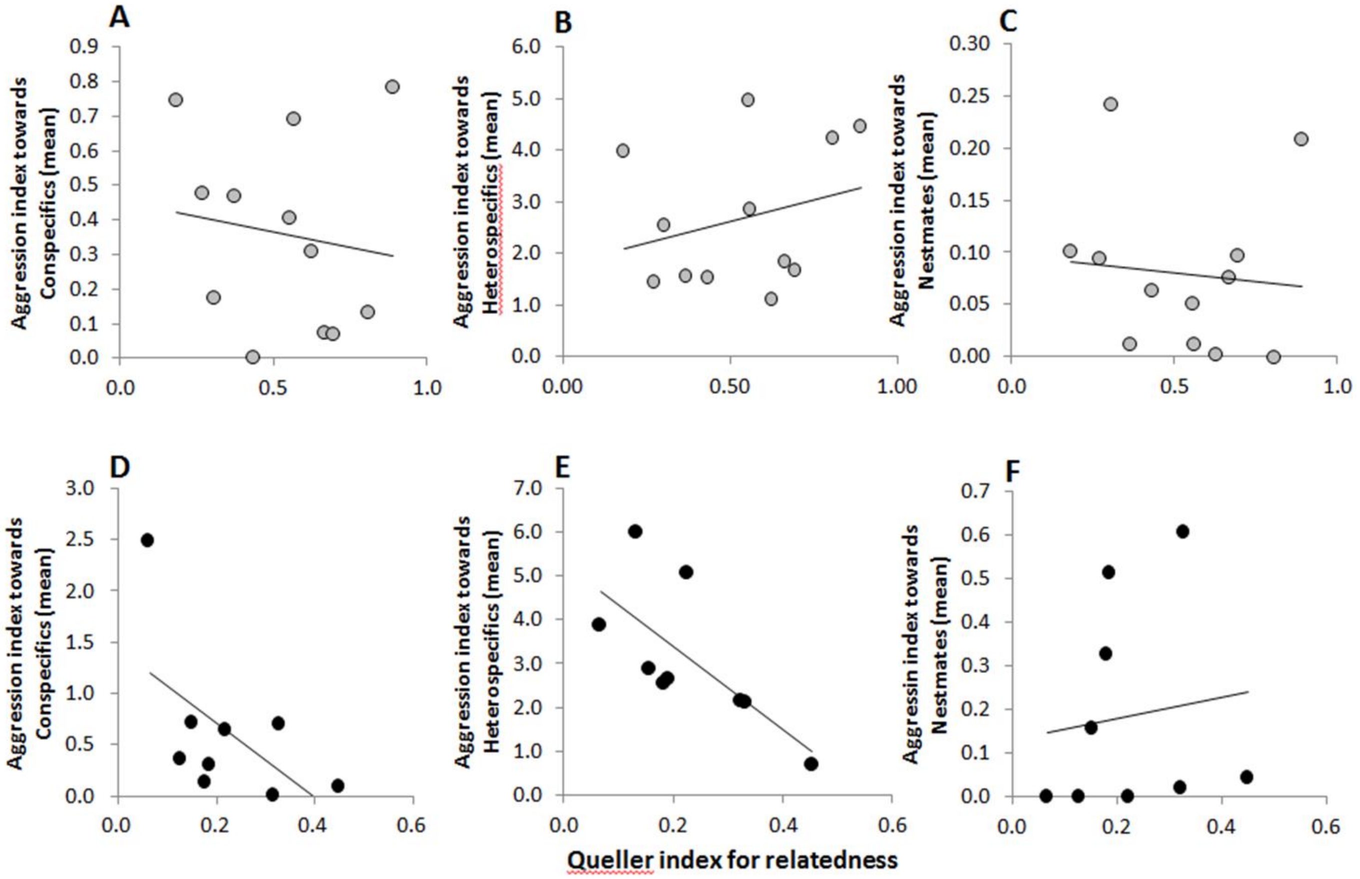

Figure S4: Correlations between the foraging performance (average mass of the collected seeds in both X axes) and nest maintenance (proportion of obstacles removed) for *M. arenarius* (A) in grey dots and *M.* sp. (B) in black dots. Both correlations are not significant:

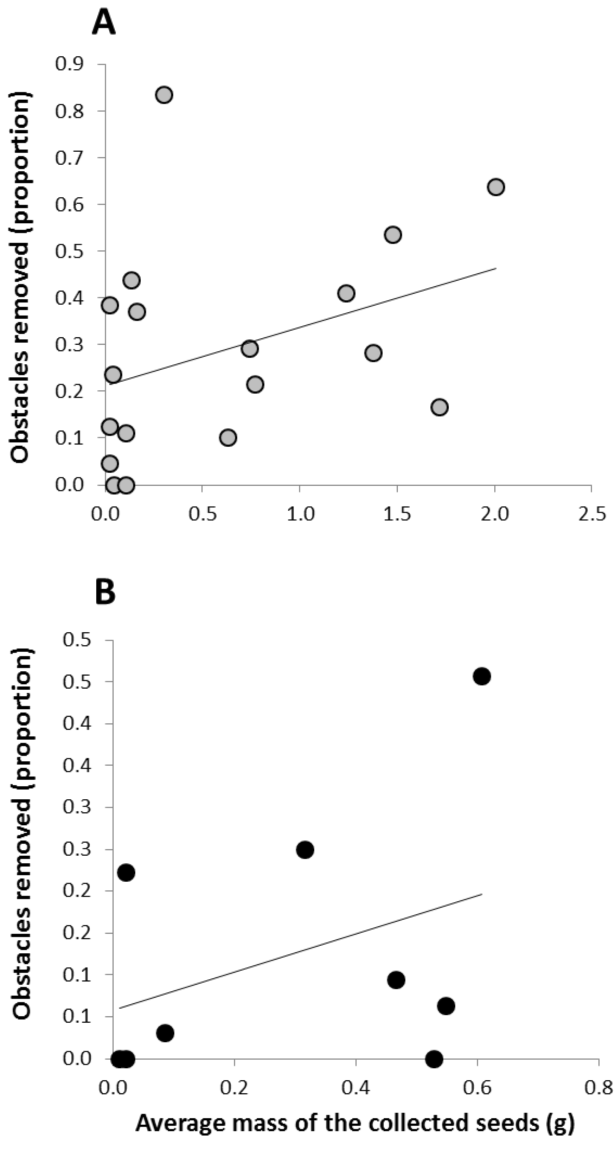

References

Chapuis M.P. & Estoup A. (2007). Microsatellite Null Alleles and Estimation of Population Differentiation. *Molecular Biology and Evolution.* 24, 621-631.

Dempster A.P., Laird N.M. & Rubin D.B. (1977). Maximum likelihood from incomplete data via the 668 EM algorithm. *Journal of the Royal Statistical Society. Series B (Methodological).* 39, 1-38.

Holland M.M. & Parson W. (2011). GeneMarker® HID: A Reliable Software Tool for the Analysis of Forensic STR Data. *Journal of Forensic Sciences*. 56, 29-35.

Rousset F. (2008). Genepop'007: a complete reimplementation of the Genepop software for Windows and Linux. *Molecular Ecology Resources*. 8, 103-106.
